# When Feedback Backfires: Effects of Real-Time Participation Feedback and Group Norm Prompt on Team Creativity in Virtual Workspaces

**DOI:** 10.3390/bs16020204

**Published:** 2026-01-30

**Authors:** Woonki Hong, Heajung Jung

**Affiliations:** School of Business Administration, Konkuk University, 120 Neungdong-ro, Gwangjin-gu, Seoul 05029, Republic of Korea; woonki@konkuk.ac.kr

**Keywords:** virtual collaboration, metaverse, team creativity, real-time feedback, group norm prompt, autonomy

## Abstract

This study examines how structured interventions influence team creativity on a metaverse-based collaboration platform. Using B.sket, a custom virtual workspace, we tested two interventions during an online brainstorming task: (1) real-time participation feedback delivered as a communication barcode showing each member’s speaking time and sequence (an informational cue), and (2) a group norm communication encouraging equal participation (a social-normative cue). Eighty-one university students in South Korea, recruited through online advertisements using a convenience sampling method, participated in a 2 (group norm prompt: provided vs. not) × 2 (participation feedback: provided vs. not) between-subject factorial design. Team creativity was evaluated by fluency, flexibility, and originality. Results revealed that, contrary to expectations, participation feedback significantly reduced idea fluency and showed marginally negative effects on flexibility and originality. The group norm prompt produced no significant improvements in creativity. We speculate that these findings can be explained by self-determination theory and ego depletion theory, such that real-time participation feedback may undermine individuals’ sense of autonomy and induce cognitive distraction, thereby reducing creative performance. We discuss practical implications that team process interventions for promoting equal participation should be designed carefully to avoid these unexpected consequences.

## 1. Introduction

As organizations increasingly adopt flexible work arrangements, an important question emerges: Will employees continue to commute to physical offices to collaborate and communicate with colleagues, or will most work be conducted remotely through online or virtual platforms? This question extends beyond logistical concerns to a more fundamental issue: Can collaboration in virtual environments match—or even surpass—the productivity and innovation typically observed in offline, co-located collaboration? Furthermore, how can organizations effectively harness the unique features of virtual spaces to facilitate more active and effective collaboration among employees?

Past research on organizational collaboration has predominantly focused on face-to-face interactions within physical environments (Duncan & Fiske, 1977). However, the evolution of digital communication technologies and the recent emergence of virtual spaces have fundamentally transformed how organizations operate. Recent research indicates that digital collaboration technologies reshape team dynamics by improving information sharing and decision-making (Lane et al., 2024), requiring virtual teams to navigate the paradoxes of balancing unity and diversity (Luciano et al., 2025). As the boundaries between physical and digital workplaces blur, this transformation challenges researchers and practitioners to rethink how collaboration and coordination are orchestrated in digital workspaces (Brucks & Levav, 2022; Handke et al., 2024).

The significance of this transformation cannot be overstated. The traditional workplace model, characterized by centralized offices and physical proximity, has defined organizational structure for over a century. However, emerging evidence suggests that this paradigm may be increasingly obsolete. In particular, the COVID-19 pandemic served as an unprecedented natural experiment, forcing a rapid and comprehensive transition from physical offices to online platforms and virtual workspaces across industries and geographies (McPhail et al., 2024). Rather than the anticipated productivity decline, empirical evidence revealed that many sectors maintained or even enhanced their operational efficiency under these distributed and digitally mediated work arrangements (Pabilonia & Redmond, 2024). As immersive technologies like virtual workspace advance, they present both new collaborative opportunities and implementation challenges for organizations (Darvish et al., 2024).

Furthermore, the increasing entry of digital natives into the workforce is further accelerating the demand for virtual collaborative work environment. The emerging workforce demonstrates a strong preference for hybrid and flexible work arrangements that align with their emphasis on work–life balance and autonomy (Osorio & Madero, 2025; Colbert et al., 2016). Raised in an environment saturated with digital communication technologies, these employees bring a fundamentally different orientation toward work and interpersonal interaction. Communication through social media, video-based tools, and immersive digital environments has become deeply embedded in their daily routines, allowing them to cultivate professional relationships and collaborate across virtual spaces without the need for physical proximity.

In this context, South Korea serves as an ideal testbed to examine the virtual work behaviors of Generation Z. Korean digital natives exemplify the generation’s high digital fluency, having historically demonstrated a proactive attitude toward adopting new media (Keum, 2010) and actively engaging with metaverse platforms like Roblox (Baek & Ha, 2023). By targeting this demographic—who are already accustomed to navigating multifaceted digital environments—this study minimizes the confounding effects of technological unfamiliarity. This allows for a clearer examination of virtual collaboration dynamics and creative processes themselves, rather than technical hurdles, offering robust insights applicable to the future global workforce.

## 2. Literature Review and Hypotheses

### 2.1. Importance of Teams and Equal Participation

Teams have long been recognized as engines of creativity and innovation within organizations (Hülsheger et al., 2009). However, research consistently demonstrates that teams underutilize their collective intellectual resources, with a critical barrier being the unequal distribution of participation among team members (Gibbs et al., 2021). From the perspective of Information Processing Theory, teams function as cognitive systems that distribute, process, and integrate information. Equal or balanced participation serves as a precondition for accessing team members’ full creative potential because it exposes team members to diverse perspectives and cognitive stimulation that enhance creative problem-solving (Park & DeShon, 2010; Strauß & Rummel, 2021). Research on hidden profiles demonstrates that teams frequently fail to discuss and integrate information held uniquely by individual members (Lu et al., 2012). This information loss occurs partially because quieter or lower-status members do not contribute their distinctive knowledge, while dominant voices shape the discussion toward already-known commonalities.

The relationship between participation equality and creativity operates through several mechanisms. First, balanced participation exposes team members to more diverse perspectives and cognitive stimulation (De Dreu & West, 2001). When all team members contribute equally, the group accesses a richer repository of ideas, experiences, and viewpoints. This cognitive diversity has been repeatedly shown to enhance creative problem-solving and innovation. Second, equal participation distributes cognitive load more evenly across team members, reducing fatigue in dominant contributors and enabling sustained creative thinking throughout discussions (Paletz & Schunn, 2010). Third, equitable participation patterns signal to quieter members that their contributions are valued, strengthening their psychological safety and participatory efficacy—critical prerequisites for creative ideation (Huang & Liu, 2021).

Despite the theoretical and empirical evidence for equal participation, teams face substantial obstacles in achieving such balance. Social dynamics within teams—including status hierarchies, personality differences, and cultural norms—naturally produce participation imbalance (Gould, 2002; Lichtenstein et al., 2004). Individuals with higher status or extraversion tend to dominate discussions, while anxious or introverted members withdraw, perpetuating cycles of inequality that undermine team creative potential.

Self-Regulation Theory offers further insight into why these imbalances persist. According to this theory, effective behavioral regulation requires a feedback loop where individuals monitor their current behavior against a standard. However, teams lack adequate mechanisms for monitoring participation patterns in real time (Glikson et al., 2019). Without awareness of who is speaking and for how long, team members rely on impressions that often differ from actual participation. This lack of monitoring makes it difficult for teams to notice participation imbalances and to develop norms that support more balanced contribution.

### 2.2. Prior Research on Team Process Interventions

Recent advances in digital technologies and behavioral interventions offer promising approaches to enhance equal participation (Park et al., 2023). Behavioral feedback loops represent one such innovation, wherein systems analyze user behavior in real time and deliver immediate feedback on behavioral quality (Porter & Grippa, 2020). Real-time visual feedback on participation provides team members with objective, moment-to-moment information about their own and peers’ contribution rates. This feedback addresses the awareness gap, triggers sense-making processes about participation norms and expectations, and activates regulatory mechanisms to adjust behavior toward greater balance (Pifarré et al., 2014).

A recent study demonstrated that real-time feedback on participation significantly boosted participatory efficacy, which served as a mediating mechanism that improved both the quality and quantity of creative ideas (Park et al., 2023). Complementing this, Damian et al. (2016) found that behavioral feedback loops fostered more balanced discussions by reducing disparities in speaking duration among group members. Furthermore, Konradt et al. (2015) highlighted that combining feedback with guided reflexivity was more effective than either intervention alone, leading to superior team performance through enhanced shared mental models and team adaptation.

While previous studies have examined real-time feedback systems (Petryk et al., 2022; Rivera et al., 2021) and guided reflexivity interventions separately or in combination (Konradt et al., 2015), important research gaps remain. Most prior research has focused on either technological feedback alone or reflexivity interventions without examining how explicit verbal norm prompt operates as an independent mechanism. Furthermore, few studies have directly compared the independent and relative effectiveness of technological feedback versus normative communication approaches. Additionally, existing research has been conducted primarily in traditional face-to-face or synchronous online environments (Cortez et al., 2009), with limited examination in emerging virtual spaces such as metaverse platforms (Maslova et al., 2025) where social cues are mediated through avatars and interaction dynamics may differ substantially. This study addresses these gaps by (1) comparing two distinct intervention approaches—real-time visual feedback and explicit verbal norm communication—allowing assessment of their independent and potential synergistic effects on team creative performance, and (2) testing these interventions within a metaverse environment, providing insights into how participation-focused interventions function in next-generation virtual collaboration spaces where traditional nonverbal cues are substantially attenuated.

### 2.3. The Effects of Team Process Interventions on Team Creativity

**Figure 1:**
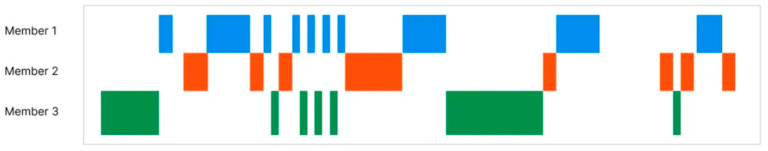
Real-time visual feedback of team interaction (“communication barcode”). *Note*. The communication barcode visualizes each member’s speaking duration and sequence, enabling the identification of interaction patterns such as turn-taking, speaking dominance, silence, and the overall within-group distribution of participation. Colors are used to distinguish individual speakers. The visualization updates in real time.

To explore ways to promote equal participation among team members and enhance team performance (e.g., creative outcomes) in virtual workspaces, this study examines the effects of two specific interventions during a team creativity task in a virtual platform. The first intervention provides an informational cue by offering participants a ‘communication barcode’—a real-time visual feedback tool that displays speaking time and interaction flow in a barcode-like format (see Figure 1). This tool is designed to enhance participants’ awareness of their contribution patterns, thereby encouraging more balanced participation across team members. Such equilibrium in interaction is critical for fostering a psychologically safe environment where diverse perspectives can be expressed and considered (Edmondson, 1999; Park & DeShon, 2010).

Real-time visual feedback on participation rates operates through several interconnected theoretical pathways to enhance team creative performance (Park et al., 2023). When team members receive moment-to-moment information about participation patterns displayed visually, they become consciously aware of imbalances between their own and others’ contributions and can interpret feedback in relation to equality norms. This awareness activates sense-making processes (Schildt et al., 2019) that help members understand the meaning of observed patterns and adjust their behavior accordingly. As team members receive feedback and successfully regulate their participation in response, they develop a stronger sense of efficacy specific to group participation (e.g., participatory self-efficacy) through direct experience of behavioral control and successful contribution (Park et al., 2023; Tolli & Schmidt, 2008).

Members with enhanced participatory self-efficacy become more motivated to engage in idea generation and creative problem-solving, translating this heightened motivation into higher team creative performance. Park et al. (2023) demonstrated that individuals receiving interactive feedback experienced increased participatory efficacy, which subsequently predicted higher individual creativity. Similarly, Damian et al. (2016) found that behavioral feedback loops produced more balanced group discussions by reducing participation imbalance.

**Hypothesis** **1.** *Teams receiving real-time visual participation feedback (e.g., communication barcode) during group discussions will show higher team creativity compared to teams not receiving such feedback.*

The second intervention examines a social-normative cue through explicit verbal instruction, which establishes a group norm emphasizing equitable participation during team collaboration. Such explicit norm-setting shapes participants’ expectations around behavior during collaboration. Group norms are powerful mechanisms for guiding interaction, especially in virtual environments where informal social cues may be weak or ambiguous (Chatman & Flynn, 2001). By instructing participants to share airtime and engage equally, the intervention promotes inclusive communication, enabling less dominant or minority voices to be heard (De Dreu & West, 2001; Nishii, 2013).

While real-time feedback addresses the awareness dimension of participation imbalance, verbal group norm prompt operates through expectancy clarification and goal-setting mechanisms. When norms regarding equal participation are explicitly communicated before team activities begin, this establishes shared expectations about appropriate behavior and signals organizational support for inclusive contribution.

These verbal communications accomplish several functions relevant to team creative performance. First, normative communication provides an interpretive frame through which team members understand subsequent feedback and behavioral adjustments (Smith & Louis, 2008). By establishing that equal participation is valued and normative, explicit communication transforms what might otherwise appear as self-serving or defensive participation regulation into prosocial contribution toward team goals. This reframing reduces potential embarrassment or defensiveness when team members recognize imbalances in their own participation, making them more willing to adjust their behavior.

Second, normative communication strengthens the intention–behavior link. Research on the theory of planned behavior demonstrates that intentions are imperfectly translated into action, with gaps arising from competing intentions, environmental constraints, and implementation failures (Webb & Sheeran, 2006; Fife-Schaw et al., 2007). However, when norms are explicitly established and internalized, behavioral intentions crystallize into stronger, more actionable commitments. Team members who have heard explicit equal participation norms are more likely to notice participation imbalances during discussion and actively adjust their participation to correct them.

**Hypothesis** **2.** *Teams receiving verbal communication of equal participation norms before engaging in group discussions will show higher team creativity compared to teams not receiving such normative communication.*

## 3. Method

### 3.1. Development of Metaverse Platform for Experimental Research

The Metaverse is defined as a post-reality universe, a perpetual and persistent multi-user environment merging physical reality with digital virtuality (Mystakidis, 2022). It offers an immersive, multisensory experience that enables real-time, embodied interaction, enhancing the sense of “being there” or social presence among remote users.

Recent scoping reviews highlight that metaverse platforms are particularly effective for collaborative learning and interaction due to their specific affordances (Sharma et al., 2023). Key features that facilitate this collaboration include the following: (1) Avatar-based representation, which allows users to project their identity and engage in non-verbal communication; (2) Synchronous interactivity, enabling real-time voice and gesture-based exchanges; and (3) Spatial navigation, which permits users to move freely and organize themselves in a virtual 3D space. These features collectively foster a sense of co-presence and psychological engagement, which are critical for creative teamwork in virtual environments.

Building on these theoretical foundations, we developed a custom metaverse-based platform named B.sket for experimental research designed to examine team interactions and assess the effects of the interventions in virtual environments. B.sket, short for “Behavior Sketch,” embodies the idea of visualizing and tracking interaction patterns and behavioral data in real time. Beyond basic video conferencing, the platform incorporates multiple features that enable rich, multimodal interaction—facilitating both verbal and non-verbal communication in immersive virtual spaces.

**Figure 2:**
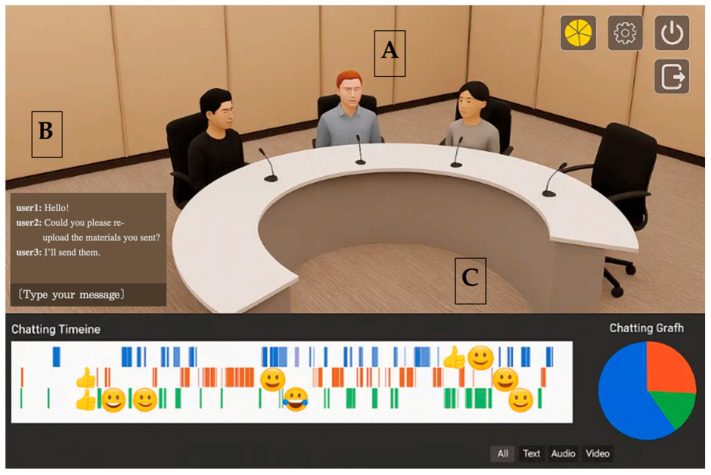
User Interface and Interaction Features of the B.sket Platform. *Note.* The platform interface consists of three key functional zones: (A) Virtual Meeting Space: Participants are represented by customizable avatars seated at a round table, facilitating a sense of co-presence and spatial awareness. (B) Multimodal Communication Panel: Located on the left, this panel supports synchronous text chatting and displays discussion history, complementing real-time voice communication. (C) Real-Time Participation Feedback System: The bottom section visualizes speaking activity in real time. The “Communication Barcode” (timeline) displays the sequence and duration of each member’s speech, while the Pie Chart on the right summarizes the cumulative speaking proportion. This visual feedback served as the informational cue for the experimental intervention.

The key functions of B.sket include video exposure through webcam for sharing live facial expressions and nonverbal cues, avatar creation and customization for personalized virtual representation, free movement and gestural interaction enabling actions like walking and waving to enhance presence, emotion expression through real-time emoji displays, and multimodal communication tools supporting both text chat and voice communication (See Table 1 and Figure 2 for details). B.sket provides a comprehensive virtual environment where participants can navigate their avatars through three-dimensional spaces while engaging in meaningful interactions with other users. This platform serves as both an experimental space for studying virtual collaboration and a data collection system for analyzing behavioral patterns in digital environments.

### 3.2. Sample and Procedure

Participants were undergraduate and graduate students enrolled at universities in South Korea. Recruitment was conducted by posting an experimental announcement on an online platform targeted at university students, which constitutes a convenience sampling method. Although this sampling method limits the representativeness of the sample, our study employed an experimental research methodology involving random assignment of participants to different conditions. This procedure is expected to minimize systematic differences between conditions, reducing the likelihood that observed effects were driven by individual differences rather than the experimental manipulation. A total of 116 students (30 teams) initially registered for the experiment. During the data screening process, teams were excluded if they encountered technical issues that interfered with task completion or if team members failed to follow key procedural requirements, such as completing both the pre-survey and the post-survey. After these exclusions, the final sample consisted of 81 participants (Mage = 24.05, 59.3% female) across 23 teams who successfully completed both the brainstorming task and all required survey measures.

Students interested in participating signed up for a preferred time slot and, upon confirmation, received an informed consent form and a pre-survey via email. Prior to the scheduled session, participants were instructed to install the B.sket application on their personal computers to ensure technical readiness. At the scheduled time, participants logged into the B.sket platform and were first prompted to create and customize their avatars by selecting features such as facial appearance, hairstyle, and clothing, enabling individualized virtual embodiment. After completing the avatar setup, participants navigated their avatars into a designated virtual meeting room as instructed. Once inside the virtual space, the experimenter provided a brief overview of the experimental procedures and conducted technical checks to verify the proper functioning of each participant’s microphone and camera before initiating the session.

Participants were assigned to teams of three to four members depending on sign-ups for each time slot and took part in a 20 min team-based brainstorming session. The task required each group to generate as many creative ideas as possible in response to the following prompt: to develop an original and compelling slogan aimed at attracting more international exchange students to Korea (Choi et al., 2019). Participants were encouraged to communicate freely and collaboratively, and each team submitted a compiled list of their ideas at the end of the session. Following the brainstorming activity, participants completed a post-experiment questionnaire. Each participant received KRW 20,000 as compensation upon successful completion of all study components.

### 3.3. Experimental Design

To test our research questions, we manipulated two key variables: (1) whether a group norm prompt emphasizing fair participation was provided, and (2) whether participants received real-time visual feedback—termed a communication barcode—that displayed how much each member was speaking during the task (see Figure 1). Participants were randomly assigned to one of four experimental conditions derived from a 2 (group norm prompt: provided vs. not provided) × 2 (real-time participation feedback: provided vs. not provided) between-subject factorial design shown in Table 2. Each condition was designed to manipulate the social-normative and informational cues that could influence group interaction during the brainstorming task.

Condition 1 combined both real-time participation feedback (informational cue) and group norm prompt (social-normative cue). Participants received an explicit verbal instruction (norm-setting) from the experimenter emphasizing the importance of equitable participation among team members. They were encouraged to ensure that everyone had equal opportunities to contribute during the session. In addition, they received real-time feedback in the form of communication barcode—a visual representation of each participant’s speaking time. The barcode was displayed throughout the discussion. This condition was intended to simulate a high-awareness, high-normative environment that supports both motivational and self-regulatory mechanisms for collaborative behavior.

Condition 2 involved group norm prompt without the real-time participation feedback via communication barcode. Participants were verbally instructed to strive for balanced and fair participation, but no visual feedback regarding speaking time was provided. This condition allowed for the isolation of normative influence without real-time informational support.

Condition 3 included the real-time participation feedback but omitted group norm prompt. In this setting, participants received no instruction about fairness or equal participation. However, they could still observe a real-time communication barcode indicating each team member’s speaking duration. This condition was designed to test whether feedback alone—absent normative framing—could shape participation dynamics.

Condition 4 functioned as the control group. Participants received neither group norm instructions nor the communication barcode. Brainstorming in this condition proceeded naturally, without any structured intervention, thereby offering a baseline for comparison against the other experimental manipulations.

### 3.4. Team Creativity

Team performance was evaluated using three commonly used indicators of creativity: fluency, flexibility, and originality (Nijstad et al., 2010). Fluency was operationalized as the total number of ideas generated by each team. A higher number of ideas was considered indicative of greater creative productivity. Flexibility captured the breadth of conceptual categories represented in a team’s ideas. The number of categories covered by a team’s output served as an index of the team’s cognitive flexibility—that is, their ability to approach the problem from multiple perspectives. Originality was evaluated qualitatively, reflecting how novel, uncommon, or innovative each idea was. Four graduate students were trained to code the responses. First, they reviewed and analyzed all the slogans generated by the participants and generated eight conceptual categories (e.g., Korean culture, exchange student benefits, diversity, etc.) for the flexibility coding. Next, the coders received detailed definitions and coding rules for each indicator, independently coded a set of 10 sample responses, and engaged in calibration sessions to align their interpretations. Once the coding scheme had been established, the raters individually coded the brainstorming outputs from each team, and the average scores across raters were used as the final fluency, flexibility, and originality scores for each team.

## 4. Results

### 4.1. Manipulation Checks

To assess manipulation effectiveness, participants indicated whether they noticed the real-time participation feedback (“communication barcode”) during the team activity and whether they perceived an equal-participation group norm established by the experimenter. A 2 × 2 χ^2^ test showed that those assigned to participation feedback conditions were more likely to report receiving feedback than those not assigned (83.3% vs. 17.9%), χ^2^(1, N = 81) = 34.63, *p* < 0.001, φ = 0.65. For the group norm prompt, participants in the norm condition were also more likely to perceive equal-participation norm than those not assigned (25.6% vs. 9.5%), χ^2^(1, N = 81) = 3.67, *p* = 0.055 φ = 0.21 (marginal).

Importantly, the weaker second effect likely reflects our manipulation check item: we asked whether a norm had been established (i.e., perceived emergence of a group norm), rather than whether participants received an explicit instruction from the experimenter. Perceived norm formation is a higher bar than instruction receipt, which can attenuate detection of the manipulation.

### 4.2. Effects of Two Interventions on Team Creativity

First, to test our hypotheses, we conducted independent-samples *t*-tests. The results indicated that neither intervention produced the expected benefits, leading to the rejection of both Hypotheses 1 and 2. Contrary to our Hypothesis 1, providing participants with real-time participation feedback via communication barcode—a visual feedback tool that displayed individual speaking time during group discussions—significantly decreased creativity outcomes, particularly in idea fluency (See Table 3). Idea Fluency (number of ideas generated) was significantly higher in the conditions without real-time participation feedback (Condition 2 + 4: M = 12.13, SD = 7.15) compared to when it was provided (Condition 1 + 3: M = 6.96, SD = 3.07), with a statistically significant difference (*p* = 0.032, Cohen’s *d* = 0.956). Differences in idea flexibility (range of idea categories) and idea originality (novelty of ideas) also favored the no real-time feedback condition, though these were marginally significant (*p* = 0.078 and *p* = 0.067, respectively). These results suggest that real-time visual feedback about one’s own and others’ participation, rather than promoting balance and creativity, may have introduced pressure, distracted attention, or undermined autonomy, ultimately disrupting the creative flow in brainstorming sessions.

Regarding Hypothesis 2, our expectation that highlighting a group norm emphasizing equitable participation would enhance team creativity was not supported by the data (See Table 4). Across all creativity indicators (i.e., fluency, flexibility, and originality), there were no statistically significant differences between the group norm prompt and no prompt conditions (all *p*-values > 0.05). Consistent with these findings, a two-way general linear model (Participation Feedback × Group Norm Prompt) indicated no significant interaction effects on any creativity indicator (all *p*s > 0.05), and the main effect of the group norm prompt was non-significant. One possible explanation is that a brief and externally imposed instruction about fair participation did not establish a psychologically meaningful group norm within the limited interaction time. Without deeper social bonding or mutual commitment, the instruction alone may have lacked the motivational force necessary to shape collaborative behavior in a metaverse environment.

Among the three team creativity indicators (fluency, flexibility, and originality), statistically significant effects were observed exclusively for Idea Fluency. These findings were corroborated by a subsequent 2 (Feedback) × 2 (Group Norm) ANOVA, which confirmed a significant main effect of Participation Feedback on Idea Fluency (F(1, 19) = 4.745, *p* < 0.05). Again, contrary to our expectations, the direction of this effect was opposite to Hypothesis 1. The analysis also revealed that neither the main effect of Group Norm (F < 1, ns) nor the interaction effect (F(1, 19) = 1.107, ns) was statistically significant. Although the interaction was not significant, a simple main effects analysis was conducted to examine the locus of the feedback effect. The results showed that this significant difference was specifically observed under the no group norm prompt condition (*p* < 0.05), but not under the group norm prompt provided condition (*p* > 0.05).

To verify the robustness of these findings given the small sample size (N = 23), we performed bootstrapping (5000 resamples). The 95% bias-corrected confidence interval for the feedback effect did not include zero (95% CI [0.475, 9.866]). Additionally, a Mann–Whitney U test yielded consistent results (U = 33.5, *p* < 0.05). Consequently, Hypothesis 1 was not supported, as the significant difference was observed in the direction opposite to our prediction.

### 4.3. Additional Analyses

To assess whether team gender composition varied systematically across our experimental conditions and to explore its potential influence on our main outcomes, we conducted an additional set of analyses. Firstly, an Analysis of Variance (ANOVA) was performed to compare the gender composition (operationalized as the percentage of female members) across the four experimental conditions. This analysis indicated no statistically significant differences in gender composition among the conditions (F(3, 19) = 0.266, *p* = 0.849), confirming successful randomization with respect to this demographic variable.

Secondly, to examine the direct and interactive effects of gender composition, we incorporated it as a fixed-effect covariate into our mixed-effects model for participation dynamics. The results showed that gender composition did not have a significant main effect on team participation dynamics (F(5, 13) = 0.619, *p* = 0.688). Moreover, the interaction effect between gender composition and the experimental conditions was also found to be non-significant (F(3, 13) = 0.943, *p* = 0.448). These findings suggest that the observed effects of our structured interventions on team creativity and participation dynamics were robust and not significantly influenced or moderated by the gender composition of the teams in this study.

## 5. Discussion

This study investigated two interaction-based interventions: (1) real-time participation feedback delivered as a “communication barcode, which provided real-time visual feedback on individual speaking time, and (2) a group norm prompt, which encouraged equitable participation. We argued that such interventions could enhance team creativity in a metaverse-based collaborative task. Contrary to our hypotheses, neither intervention significantly improved creative performance. Most strikingly, the participation feedback intervention significantly reduced idea fluency (M = 6.96 vs. 12.13, *p* = 0.032, Cohen’s *d* = 0.956), with consistent negative trends across idea flexibility and originality. This suggests that visual self-monitoring of participation may have inadvertently constrained idea generation, possibly by inducing self-consciousness or narrowing focus on turn-taking rather than idea elaboration. Similarly, the group norm prompt emphasizing equal contribution did not yield a statistically significant effect, indicating that verbal reminders alone may be insufficient to alter ingrained participation patterns in short-term virtual interactions.

These findings underscore the complex and sometimes counterintuitive effects of feedback and norms in digitally mediated collaboration. While equitable participation is often assumed to promote creativity, its facilitation in immersive virtual environments requires careful consideration of how social cues are perceived and cognitively processed. Importantly, the results offer key lessons for designing more effective virtual teamwork environments.

### 5.1. Autonomy and the Cognitive Cost of Feedback

Self-determination theory (Ryan, 1982; Deci & Ryan, 2000) provides a useful framework for understanding why real-time participation feedback reduced creativity. The theory posits that feedback perceived as controlling—rather than informational—undermines intrinsic motivation by threatening autonomy. In our study, the combination of explicit instructions (e.g., “everyone should speak equally”) and real-time participation feedback through the communication barcode may have inadvertently threatened participants’ sense of autonomy (e.g., Enzle & Anderson, 1993). This monitoring likely triggered a shift in cognitive focus. Rather than fostering spontaneous idea generation, participants may have shifted their attention toward regulating how much they were speaking—asking themselves whether they were contributing “enough” or “too much” compared to others. As a result, the intervention may have disrupted the fluidity and associative thinking processes that are essential for effective brainstorming, ultimately impairing the quantity of ideas generated. While effects on idea flexibility and originality were marginal (*p* = 0.078 and 0.067), the consistent negative direction across all creativity dimensions suggests this mechanism may broadly affect creative performance, though future research with larger samples is needed to confirm these patterns.

### 5.2. Ego Depletion and Self-Regulatory Overload

This interpretation is further supported by research on ego depletion (Baumeister et al., 2018), which suggests that tasks requiring constant self-control—especially when people feel they are being watched—can drain the mental resources needed for subsequent cognitive performance. In our study, participants exposed to the communication barcode may have experienced self-regulatory fatigue, as they were constantly calibrating their speech to maintain “equal participation,” instead of focusing on generating original ideas. Importantly, reduced autonomy and self-regulatory overload should not be viewed as independent explanations. Rather, autonomy threat may have triggered heightened self-monitoring, which in turn imposed ongoing self-regulatory demands, crowding out the attentional and cognitive resources required for creative idea generation. This could lead to reduced attentional focus, lower motivation, and even self-licensing (“I spoke enough, so I’ve done my part”), all of which are detrimental to creative outcomes.

### 5.3. Challenges of Group Norm Interventions Among Strangers

The second intervention—framing equal participation as a group norm—failed to produce significant improvement in team creativity. While we must be cautious in drawing strong theoretical conclusions from null findings, one plausible explanation is the limit of salience and reinforcement. A single verbal prompt at the beginning of the task may not be sufficient to establish shared norms, especially in short-term groups of strangers with no prior interaction. Effective group norms are typically shaped through ongoing interpersonal exchanges, developing mutual trust, and alignment around shared goals—conditions that were absent in our experimental setting (e.g., Kim & Shin, 2015; Postmes et al., 2000). This limitation may be especially pronounced in virtual environments where social cues are attenuated and normative signals lack the interpersonal reinforcement commonly present in face-to-face interaction. As a result, externally imposed norms may remain cognitively acknowledged but behaviorally weak. However, it is also possible that our sample size limited statistical power to detect small effects, or that the norm prompt requires specific conditions (e.g., longer interaction time, established teams) to be effective. Future research should explore these boundary conditions before concluding that norm prompts are ineffective in virtual settings.

## 6. Practical Implications

Although this study aimed to enhance collaboration and creativity in a metaverse-based team environment through structured interventions, the findings revealed unintended and even counterproductive outcomes. Instead of promoting engagement, the interventions—particularly the real-time participation feedback and group norm prompt—appeared to reduce participants’ sense of autonomy, depleting cognitive resources and weakening intrinsic motivation. Yet, these unexpected outcomes offer important lessons for designing future virtual collaboration environments.

### 6.1. Not Controlling, but Nudging

The results underscore a critical lesson: interventions in virtual workspaces should support, not undermine, users’ sense of autonomy. Digital platforms, especially those operated within organizational settings, often have powerful data collection capabilities—tracking login times, chat activity, or even avatar movement within virtual offices. While these features can provide useful real-time feedback, they risk being perceived as tools of micromanagement or surveillance, which may provoke resistance and reduce engagement (Douthitt & Aiello, 2001; Giacosa et al., 2023).

Instead of prescriptive or evaluative interventions, platforms should aim to foster self-awareness through subtle, autonomy-supportive cues. Information about team dynamics or communication patterns can be helpful when presented as reflective prompts rather than corrective mandates. For instance, a real-time communication barcode may seem beneficial in theory, but if overly emphasized, it can lead to mental exhaustion from constantly checking one’s own behavior. This pressure makes users less likely to act or speak naturally on impulse, which ultimately stifles creative engagement. A more effective approach may involve subtle nudges that respect individual autonomy while promoting team-level awareness.

### 6.2. Less Intrusive, More Playful Feedback

An important design question emerges: how can we encourage better collaboration without intruding on users’ cognitive resources or autonomy? During brainstorming activities in our study, participants who allocate cognitive resources to behavioral self-monitoring—such as “Am I speaking enough?”—detract from attention and motivation for creative tasks (Inzlicht & Schmeichel, 2012). Real-time behavioral feedback, despite its beneficial intentions, can inadvertently interfere with idea generation. Post-session feedback mechanisms offer a promising alternative that preserves collaborative flow while maintaining the developmental benefits of behavioral awareness. Delayed reflection allows participants to examine their interaction patterns retrospectively, when cognitive resources are no longer divided between creative generation and behavioral regulation. This temporal separation enables participants to process their collaborative experiences without the immediate pressure of ongoing performance demands.

For such feedback to be effective, it needs to be engaging and meaningful. One underexplored strength of metaverse environments is their inherent playful and immersive potential. Features such as avatars and spatial environments offer novel opportunities to design feedback that is less intrusive yet still motivating. For instance, avatar-based feedback—such as gradual changes in size, opacity, or visual prominence corresponding to engagement levels—can provide continuous behavioral cues that encourage action without enforcing it. Such gamified feedback mechanisms tap into intrinsic motivational systems while allowing users to maintain autonomy (McGonigal, 2011). Rather than prescriptive instructions or numerical performance metrics, these ambient feedback systems can function as subtle environmental cues that inform without commanding.

### 6.3. Fostering Group Norms in Virtual Teams

Establishing shared identity and group norms represents another challenge in virtual collaborative environments. In traditional teams, these typically emerge through physical proximity, shared experiences, and spontaneous interactions (Kabo, 2017; Reagans, 2011). Digital platforms, particularly metaverse environments, often lack these natural mechanisms. Our empirical findings demonstrate this limitation clearly. Explicit group norm interventions, such as instructions to “speak equally,” produced no meaningful behavioral changes among participants. This failure occurred primarily because such directives were implemented early in the collaboration session, before sufficient group cohesion had developed. The temporal constraints of virtual interactions appear insufficient for participants to internalize prescribed norms or develop genuine collective identity.

The ineffectiveness of early norm implementation reflects deeper structural issues in virtual collaboration. Recent research suggests that design elements in virtual environments can subtly influence norm formation and team identity. For instance, avatar design may impact perceived similarity and interpersonal closeness (Van Der Land et al., 2015)—raising important design questions such as: Should avatars reflect users’ real appearances, or would a shared visual style foster stronger group affiliation? Environmental cues also matter. In one study (Belmi & Schroeder, 2021), participants interacting with a colleague shown in a casual coffee shop background perceived the relationship as more personal, whereas a professional office background elicited a more transactional impression. These findings indicate that even small design cues—visual context, avatar similarity, background design—can shape how virtual team members perceive and relate to one another. However, much remains underexplored. As the nature of work continues to evolve toward remote and hybrid modalities, future research should investigate how virtual design choices can gently nudge collaboration norms without relying on overt control.

## 7. Conclusion, Limitations and Future Research Directions

This study aimed to examine how structured interventions influence team creativity in a metaverse-based collaboration platform. We introduced two specific interventions within the B.sket virtual environment: (1) real-time participation feedback via a communication barcode (an informational cue), and (2) a group norm prompt encouraging equal participation (a social-normative cue). Contrary to initial expectations, neither intervention significantly improved creative performance. Notably, the real-time participation feedback inadvertently hindered creativity, likely by harming one’s sense of autonomy and increasing cognitive load, thereby disrupting the flow required for divergent thinking. The group norm prompt also showed limited impact, possibly because verbal instructions alone may lack the reinforcement necessary to alter behavior in virtual settings without prior cohesion.

These findings offer a critical counter-narrative to the assumption that “more feedback is better.” They suggest that interventions in virtual teamwork must be carefully designed to preserve user autonomy and minimize psychological burden. The results also point to the importance of designing virtual environments—including feedback mechanisms, avatar representation, and spatial cues—in ways that gently ‘nudge’ interaction norms rather than imposing control. This research contributes to a nuanced understanding of how digital platforms shape collaboration dynamics and offers practical insights for developing metaverse-based systems that support, rather than constrain, creative teamwork.

While this study offers meaningful insights, several limitations qualify the generalizability of its findings and point to directions for future research. First, the study relied on a relatively small group-level sample size (N = 23 teams). While individual-level data were available (N = 81), we maintained the analysis at the team level to ensure statistical validity given that our dependent variables were group-level constructs. To address concerns regarding statistical power, we conducted a post hoc sensitivity analysis using G*Power (version 3.1.9.7; Heinrich Heine University Düsseldorf, Düsseldorf, Germany) for a *t*-test (one-tailed, alpha = 0.05, power = 0.80), which indicated that our design was sensitive to detecting a large effect size of *d* = 1.07. Notably, the observed effect size for our primary finding (*d* = 0.96) closely approached this threshold. Furthermore, bootstrapping analysis (5000 resamples) yielded confidence intervals that did not include zero, confirming that the observed performance differences are robust and practically significant despite the sample size constraints.

Second, our study relied on a sample of South Korean university students. We acknowledge that this may limit immediate generalizability to senior professionals or different cultural contexts. However, this demographic was strategically selected as a “testbed” for exploratory research. South Korean youth represent a “digital native” population with exceptionally high acceptance of new technologies and high familiarity with metaverse platforms. By studying this high-engagement group, we aimed to minimize confounding factors related to technological unfamiliarity, thereby isolating the psychological effects of the interventions. These participants offer forward-looking insights into how future general users might behave as metaverse adoption becomes ubiquitous globally.

Third, the experiment utilized ad hoc teams performing a single short-term brainstorming task. While this design precludes the observation of long-term group evolution, it offers a distinct methodological advantage for this experimental setup. Utilizing groups without prior history allowed us to examine the effects of real-time feedback and norm prompts without the interference of pre-existing power dynamics, relational history, or status hierarchies. This suggests that even in nascent group interactions common in modern cross-functional teams, intrusive feedback can disrupt creative performance.

Fourth, we acknowledge that our “group norm prompt” intervention, which relied on a single verbal instruction, may have lacked operational specificity. A one-time simple instruction may be insufficient to constitute a psychologically meaningful norm induction in a virtual setting. Future research should strengthen this manipulation through more robust mechanisms, such as repeated prompts, collaborative goal-setting exercises, or visible social contracts displayed within the virtual space to reinforce desired behaviors and ensure norms are effectively internalized.

Finally, this study did not include baseline assessments of participants’ inherent creative potential or communication tendencies. Although random assignment helps mitigate systematic bias, the absence of pre-task measures limits our ability to determine whether performance differences were influenced by inherent group composition variability. To address this, subsequent studies should collect and report baseline measures (e.g., creative potential, Big Five personality traits) to control for confounding variables and more precisely isolate the impact of environmental interventions. Additionally, while our random assignment procedures ensured no significant differences in gender composition across experimental conditions, and gender ratio did not emerge as a significant covariate or interact with our interventions on fluency, we recognize that gender is a significant social factor in team dynamics. Thus, we acknowledge the need for future research to systematically explore the role of gender, particularly through studies specifically designed to manipulate or directly analyze varied gender compositions.

To strengthen external validity and robustness, future research must address these boundary conditions. Subsequent studies should replicate these findings with larger and more diverse samples, encompassing various cultural backgrounds and occupational groups. Furthermore, longitudinal designs are needed to capture how teams adapt to feedback mechanisms over time and to determine whether the negative effects of monitoring attenuate as teams develop trust and cohesion. Expanding the research scope to include complex, interdependent tasks beyond brainstorming—paired with richer behavioral log data—will further clarify how virtual environment design can effectively support team innovation.

## Figures and Tables

**Table 1:** Key Features of the B.sket Platform for Virtual Team Interaction.

Function	Description
Video Exposure via Webcam	Displays users’ facial expressions in real time for non-verbal communication.
Avatar Creation and Customization	Allows participants to create and personalize avatars for virtual embodiment.
Free Movement and Gestures	Avatars can move and perform gestures (e.g., waving, sitting) to enhance presence.
Emoji-based Emotion Expression	Enables real-time display of emotional states using emoji visuals.
Multimodal Communication	Supports both real-time voice and text chat for flexible team interaction.

**Table 2:** Study Conditions by Group Norm Prompt and Real-time Participation Feedback.

	Participation Feedback Provided	Participation Feedback Not Provided
**Group Norm Prompt** **Provided**	Condition 1	Condition 2
**Group Norm Prompt** **Not Provided**	Condition 3	Condition 4

**Table 3:** Team Creativity by Presence of Real-Time Participation Feedback.

Creativity	Participation Feedback Provided (M, SD)	Participation Feedback Not Provided (M, SD)	Mean Difference	*p*-Value	Cohen’s *d*
Idea Fluency	6.96 (3.07)	12.13 (7.15)	5.17	0.032	0.956
Idea Flexibility	2.35 (0.70)	2.89 (0.67)	0.53	0.078	0.772
Idea Originality	2.38 (0.45)	2.73 (0.43)	0.35	0.067	0.808

Note. Means (M) and standard deviations (SD). Participation Feedback = real-time “communication barcode”. *p* values from two-sample tests at the team level.

**Table 4:** Team Creativity Outcomes by Presence of Group Norm Prompt.

Creativity	Group Norm Prompt Provided (M, SD)	Group Norm Prompt Not Provided (M, SD)	Mean Difference	*p*-Value	Cohen’s *d*
Idea Fluency	9.02 (1.66)	9.81 (1.87)	0.78	0.755	0.132
Idea Flexibility	2.59 (0.21)	2.63 (0.23)	0.03	0.914	0.046
Idea Originality	2.48 (0.15)	2.60 (0.13)	0.13	0.524	0.271

## Data Availability

The raw data supporting the conclusions of this article will be made available by the authors on request.
